# A pilot metabolomics study across the continuum of interstitial lung disease fibrosis severity

**DOI:** 10.14814/phy2.70093

**Published:** 2024-10-18

**Authors:** Jiada Zhan, Zachery R. Jarrell, Xin Hu, Jaclyn Weinberg, Michael Orr, Lucian Marts, Dean P. Jones, Young‐Mi Go

**Affiliations:** ^1^ Division of Pulmonary, Allergy, Critical Care and Sleep Medicine, Department of Medicine Emory University Atlanta Georgia USA; ^2^ Nutrition and Health Sciences, Laney Graduate School Emory University Atlanta Georgia USA; ^3^ Gangarosa Department of Environmental Health, Rollins School of Public Health Emory University Atlanta Georgia USA

**Keywords:** lung disease, metabolic disruption, pathway enrichment analysis, pulmonary fibrosis

## Abstract

Interstitial lung diseases (ILDs) include a variety of inflammatory and fibrotic pulmonary conditions. This study employs high‐resolution metabolomics (HRM) to explore plasma metabolites and pathways across ILD phenotypes, including non‐fibrotic ILD, idiopathic pulmonary fibrosis (IPF), and non‐IPF fibrotic ILD. The study used 80 plasma samples for HRM, and involved linear trend and group‐wise analyses of metabolites altered in ILD phenotypes. We utilized limma one‐way ANOVA and mummichog algorithms to identify differences in metabolites and pathways across ILD groups. Then, we focused on metabolites within critical pathways, indicated by high pathway overlap sizes and low *p*‐values, for further analysis. Targeted HRM identified putrescine, hydroxyproline, prolyl‐hydroxyproline, aspartate, and glutamate with significant linear increases in more fibrotic ILD phenotypes, suggesting their role in ILD fibrogenesis. Untargeted HRM highlighted pathway alterations in lysine, vitamin D3, tyrosine, and urea cycle metabolism, all associated with pulmonary fibrosis. In addition, methylparaben level had a significantly increasing linear trend and was higher in the IPF than fibrotic and non‐ILD groups. This study highlights the importance of specific amino acids, metabolic pathways, and xenobiotics in the progression of pulmonary fibrosis.

## 1 INTRODUCTION

Interstitial lung disease (ILD) encompasses a variety of inflammatory and fibrotic pulmonary disorders, often marked by symptoms of shortness of breath, cough, fatigue, and chest discomfort (Interstitial Lung Disease American Lung Association, n.d.). Pulmonary fibrosis attributable to ILD is often irreversible and progressive. ILDs may be classified in a variety of ways, though they are frequently attributed to one of the following broad categories: distinct primary diseases (e.g., sarcoidosis); environmental exposures; drugs and irradiation; connective tissue disease; and idiopathic interstitial pneumonia, including idiopathic pulmonary fibrosis (IPF) (Wijsenbeek & Cottin, 2020).

Based on the presence and degree of fibrosis, ILDs can be stratified into non‐fibrotic and fibrotic phenotypes (Wijsenbeek et al., 2022). Non‐fibrotic ILD (NF‐ILD) primarily exhibits inflammation without significant development of scar tissue, whereas fibrotic ILD (F‐ILD) is characterized by an excessive accumulation of scar tissue in the lungs, though there may be significant overlap between non‐fibrotic and fibrotic phenotypes (Wijsenbeek et al., 2022). The distinction has important clinical relevance, as NF‐ILD is generally treated with immunosuppressive therapy, while anti‐fibrotic therapy is frequently considered in F‐ILD, including IPF. IPF, long considered the poster child of F‐ILD, has no definitive etiology, and is typically associated with a progressive, irreversible decline in lung function (Wijsenbeek & Cottin, 2020). Currently, IPF has no cure, and treatments primarily focus on slowing disease progression (Idiopathic Pulmonary Fibrosis (IPF) American Lung Association, n.d.; American Lung Association, 2023). Although all ILDs have different pathophysiology, a leading hypothesis is that in later phases of seemingly different ILDs, there are similar mechanisms of disease progression: dysregulated fibrogenesis and progressive pulmonary fibrosis (Wijsenbeek & Cottin, 2020).

The application of metabolomics has emerged as an invaluable tool in understanding disease mechanisms and informing potential treatment approaches (Dettmer et al., 2007; Johnson et al., 2016). Metabolomics enables the targeted and global profiling of metabolites within a biological sample, measuring the substrates and products of metabolism that reflect essential cellular functions (Johnson et al., 2016). Previous work has used metabolomics to study mechanisms of pulmonary fibrosis predominantly through IPF and animal‐based pulmonary fibrosis models (Gaugg et al., 2019; Kang et al., 2016; Li et al., 2023; Rajesh et al., 2023; Roque & Romero, 2021; Selvarajah et al., 2021). One common pathological characteristic is the change in amino acid metabolites, including aspartate (Zhao et al., 2017), alanine (Gaugg et al., 2019), arginine (Endo et al., 2003; Kitowska et al., 2008; Mora et al., 2006), proline (Durante et al., 2001; Hesse et al., 2001; Kitowska et al., 2008; Pesce et al., 2009), glutamine (Bernard et al., 2018; Cruzat et al., 2018; Ge et al., 2018; Hamanaka et al., 2019), glutamate (Roque & Romero, 2021), putrescine (Endo et al., 2003; Mora et al., 2006; Zhao et al., 2017), spermidine (Endo et al., 2003; Mora et al., 2006; Zhao et al., 2017), hydroxyproline (Endo et al., 2003; Gaugg et al., 2019; Zhao et al., 2017) and prolyl‐hydroxyproline (Endo et al., 2003; Zhao et al., 2017). However, few studies have investigated the metabolomic profiles of clinical ILDs integratively to better understand their metabolic commonalities and differences. In addition, fewer studies stratify ILD into clinically relevant NF‐ILD and F‐ILD phenotypes, and it remains uncertain whether IPF represents a distinct clinical diagnosis or the final result of fibrogenesis from disparate insults (Noble et al., 2012). There is a clinical and pathogenetic justification of focusing on “the progressive fibrotic phenotype” in clinical research regarding ILD and IPF, a view which is currently held in contention (Wells et al., 2018).

Building upon these initial insights, in this study we aim to determine whether specific amino acids previously associated with IPF and preclinical models of pulmonary fibrosis differ between non‐ILD, NF‐ILD, non‐IPF F‐ILD, and IPF in a progressive manner using targeted metabolomics analysis. Additionally, we aim to uncover novel metabolites and pathways that could be involved in ILD development and progression using untargeted metabolomics analysis. Through this combined approach, we hope to enhance our understanding of the fibrometabolism commonalities and differences between non‐ILD and various ILD phenotypes.

## 2 MATERIALS AND METHODS

### 2.1 Study population and blood collection

We recruited 80 patients from the Emory Pulmonary Fibrosis and Rare Lung Disease Research Registry and Biorepository at Emory Clinic and Emory University Hospital, between October 2021 and June 2022. Patients were eligible for the study if they were greater than 18 years old and determined to have pulmonary fibrosis and/or rare lung disease, including various NF‐ILD, F‐ILD and IPF. Individuals evaluated in the pulmonary clinic without pulmonary fibrosis and/or rare lung disease were enrolled as disease control (Non‐ILD). Written informed consents were obtained from participants. Patients who could not provide informed consent or were pregnant or breastfeeding were excluded. Whole blood samples were obtained from patients during the clinical visit and then separated into plasma, which was stored at −80°C until future analyses. The study was approved by the Emory University Institutional Review Board (STUDY00001384). No patients were involved in the design and conduct of this research.

### 2.2 Demographic data collection

The following clinical characteristics were extracted by the research team using Emory's electronic health record system: gender, race, age at enrolment, age at diagnosis of lung disease, smoking history, body mass index (BMI) on enrolment, lung disease diagnosis, and pulmonary fibrosis phenotype (fibrotic or non‐fibrotic). Patients were considered to have a fibrotic phenotype if the most recent chest computed tomography report or review of chest imaging prior to sample collection noted the presence of (1) traction bronchiectasis, (2) traction bronchiolectasis, and/or (3) honeycombing. Individuals without any of these findings were considered to have a non‐fibrotic phenotype. ILD clinical diagnoses were determined based on chart review of ILD physician documentation. All ILD providers took part in a weekly clinical conference, and clinical diagnoses were adjudicated based on the combination of clinical, radiographic, and pathologic criteria. IPF diagnoses were based on the established ATS/ERS/JRS/ALAT clinical practice guidelines (Raghu et al., 2022).

### 2.3 High‐resolution metabolomics (HRM)

High‐resolution metabolomics profiling was conducted at Emory Clinical Biomarker Laboratory as previously described (Go et al., 2015; Tchen et al., 2022). Briefly, each plasma sample underwent the treatment of acetonitrile, and after centrifuging, liquid chromatography‐high‐resolution mass spectrometry (LC‐HRMS) using a Dionex Ultimate 3000 (Thermo Scientific) LC coupled with a Fusion mass spectrometer (Thermo Scientific). HRMS was employed to analyze the samples in triplicate. Plasma reference samples were used to account for experimental drifts. The method consisted of hydrophilic interaction liquid chromatography (HILIC) with positive electrospray ionization (ESI). Resolution was set at 120 k for a scan range of 85–1275 *m/z*. After completing the instrumental analyses, metabolic features are obtained and characterized by high‐resolution *m/z*, retention time (rt, sec), and intensity (abundance) of mass spectral peak. The raw data files were converted to mzXML format using ProteoWizard (Go et al., 2015) and further processed with apLCMS (Yu et al., 2009) and xMSanalyzer (Uppal et al., 2013). We included metabolic features detected in more than 50% of all samples and a Pearson correlation above 0.7 among triplicates. The feature intensities were median‐summarized across triplicates.

### 2.4 Metabolite confirmation and annotation

We conducted metabolite confirmation and annotation for metabolic features using an in‐house authentic chemical library, xMSannotator, and MS^2^ database matching (Uppal et al., 2017). Metabolites in plasma reference samples identified and quantified in our laboratory, using retention time and MS^2^ spectra matching authentic standard under identical experimental conditions (Liu et al., 2020), were given Level 1 confidence score according to Schymanski (Schymanski et al., 2014) and level 1 identification according to the Metabolomics Standards Initiative (MSI) (Sumner et al., 2007). These plasma reference samples were run concurrently with the study samples.

When only the retention time was available for matching authentic standard, MS^2^ database matching was performed on MetaboAnalyst (Pang et al., 2024) and MassBank of North America (MassBank of North America, n.d.), and metabolites were thus given Schymanski level 2 confidence level and MSI level 1 identification level. When retention time was not available for matching authentic standard, MS^2^ database matching was performed, and metabolites were thus given Schymanski level 2 confidence level and MSI level 2 identification level. For those targeted metabolites and other metabolites highlighted in the pathway analysis that did not have authentic standards in our library, we selected high‐confidence annotated metabolites (M + H or M + Na) from xMSannotator and used MS^2^ database matching as an alternative strategy. If no useful MS^2^ data could be obtained, annotated metabolites were given Schymanski level 4 confidence level and MSI level 2 identification level. The OrgMassSpecR R package was used to perform dot product MS^2^ similarity comparison between sample MS^2^ and experimental MS^2^ (Dodder, 2023).

For the targeted ion dissociation in MS^2^ identification, plasma samples with the highest intensity of the compounds of interest were analyzed with HILIC/ESI+. The mass spectrometer (Thermo High Field Q‐Exactive) was set to scan 50–500 *m/z* at 60 k resolution. Parallel reaction monitoring mode was used with 30 k resolution and an isolation window of 1.0 *m/z* for the targeted inclusion list. Ion dissociation was acquired in HCD mode set for 35% normalized collision energies. Raw spectra were analyzed with xCalibur QualBrowser (Thermo).

### 2.5 Statistical and pathway enrichment analyses

For demographic analysis, Fisher's exact test was used for comparing two categorical variables. The Kruskal Wallis rank sum test was used for comparing continuous and categorical variables. For targeted metabolomics analysis, the evaluation of metabolites associated with various fibrosis phenotypes was performed with linear trend analyses across the experimental groups. The exposure is an ordinal variable that represents a different stage in the spectrum of pulmonary fibrosis: non‐ILD, NF‐ILD, F‐ILD, and IPF. These groups ranged from non‐ILD, representing the lower limit or absent fibrosis, to IPF, representing definitive progressive fibrosis. Assumptions of the linear trend analyses were independence of observations, homoscedasticity, normality of residuals, and pulmonary fibrosis severity grouping was ordinal. Metabolic feature intensities were log_2_ transformed prior to analysis. To determine pairwise differences between groups, we also tested metabolite abundances by one‐way ANOVA or the Kruskal‐Wallis rank sum test followed by either Tukey's honest significant difference test or Dunn's test, respectively, depending on the distribution of each metabolite using the Shapiro–Wilk Test. Significant differences between groups are noted using asterisk notation (*p < 0.05; **p < 0.01; ***p < 0.001), and near‐significant *p*‐values are provided in figures.

To identify metabolic pathways associated with non‐fibrotic and fibrotic ILD phenotypes including IPF globally, we conducted an untargeted analysis using an unadjusted one‐way ANOVA test via the *limma* algorithm (Ritchie et al., 2015), followed by pathway enrichment analysis using the *mummichog* algorithm using metabolites with *p* < 0.05 (Sumner et al., 2007). Initially, the Benjamini‐Hochberg method for false discovery rate (FDR) correction was applied to limit FDR for metabolic feature selection at 20%; however, too few features were retained to perform pathway enrichment analysis. Because pathway enrichment analysis is itself a Type I error‐rate limiting procedure, feature selection for pathway enrichment analysis was instead performed using features selected at *p* < 0.05. For limma analysis, non‐detects were imputed using the half the minimum detected intensity and intensities were log_2_ transformed. After pathway enrichment analysis, we repeated the linear trend analysis and group‐wise comparisons for representative metabolites as described above.

## 3 RESULTS

### 3.1 Study characteristics

Plasma samples used in this study were collected from 8 non‐ILD controls, 22 with NF‐ILD, 45 with F‐ILD, and 5 IPF (Tables 1 and 2). There were significant differences in gender and BMI on enrolment between the groups. Notably, all patients in the IPF group were male and had the highest age at diagnosis of lung disease. Patients in the NF‐ILD and F‐ILD groups tended to have a higher BMI compared to the non‐ILD group. Race, age at enrollment, and smoking history were not different between groups. Specifically, the IPF group had the highest age at enrolment and the highest percentage of the population being White, and the F‐ILD group had the lowest age at enrolment.

**TABLE 1:** Demographic characteristics of study subjects.

Characteristic	Non‐ILD, *N* = 8^a^, ^c^	Non‐fibrotic ILD (NF‐ILD), *N* = 22^a^	Non‐IPF fibrotic ILD (F‐ILD), *N* = 45^a^	IPF, *N* = 5^a^	*p*‐value^b^
Gender					
Female	4 (50%)	16 (73%)	27 (60%)	0 (0%)	0.025
Male	4 (50%)	6 (27%)	18 (40%)	5 (100%)	
Race					
American Indian or Alaskan	0 (0%)	0 (0%)	1 (2.2%)	0 (0%)	0.057
Asian	0 (0%)	0 (0%)	1 (2.2%)	0 (0%)	
Black or African‐American	1 (13%)	10 (45%)	25 (56%)	0 (0%)	
White	7 (88%)	12 (55%)	18 (40%)	5 (100%)	
Age at enrolment	65 (42, 70)	59 (47, 64)	55 (49, 67)	73 (72, 76)	0.15
Age at diagnosis of lung disease	29 (19, 40)	48 (44, 60)	47 (44, 54)	72 (71, 72)	0.11
Ever smoker (cigarette, cigar)	4 (50%)	7 (32%)	15 (33%)	2 (40%)	0.8
BMI on enrolment	24 (21, 27)	29 (24, 36)	30 (28, 35)	27 (26, 29)	0.031

Abbreviations: BMI, body mass index; ILD, Interstitial lung disease; IPF, idiopathic pulmonary fibrosis.

^a^ *n* (%); median (IQR).

^b^ Fisher’s exact test; Kruskal‐Wallis rank sum test.

^c^ Non‐ILD controls included four patients with chronic obstructive pulmonary disease (two white males and two white females) and four with asthma (two white males, one black female, and one white female).

**TABLE 2:** Specific clinical diagnosis of ILD within the non‐fibrotic ILD (NF‐ILD) and non‐IPF fibrotic ILD (F‐ILD).

Clinical diagnosis	NF‐ILD, *N* = 22^a^	F‐ILD, *N* = 45^a^
Sarcoidosis	19 (86.4%)	6 (13.3%)
Connective tissue‐related ILD	1 (4.5%)	14 (31.1%)
Anti‐synthetase syndrome	1 (4.5%)	9 (20%)
Hypersensitivity Pneumonitis	0	6 (13.3%)
Interstitial pneumonia with autoimmune features	0	4 (8.8%)
Myositis associated ILD	0	3 (6.7%)
Bronchiectasis	0	2 (4.4%)
Cystic lung disease	1 (4.5%)	0
Combined pulmonary fibrosis and emphysema	0	1 (2.2%)

^a^ *n* (%); median (IQR).

### 3.2 Targeted metabolomics analyses

**FIGURE 1:**
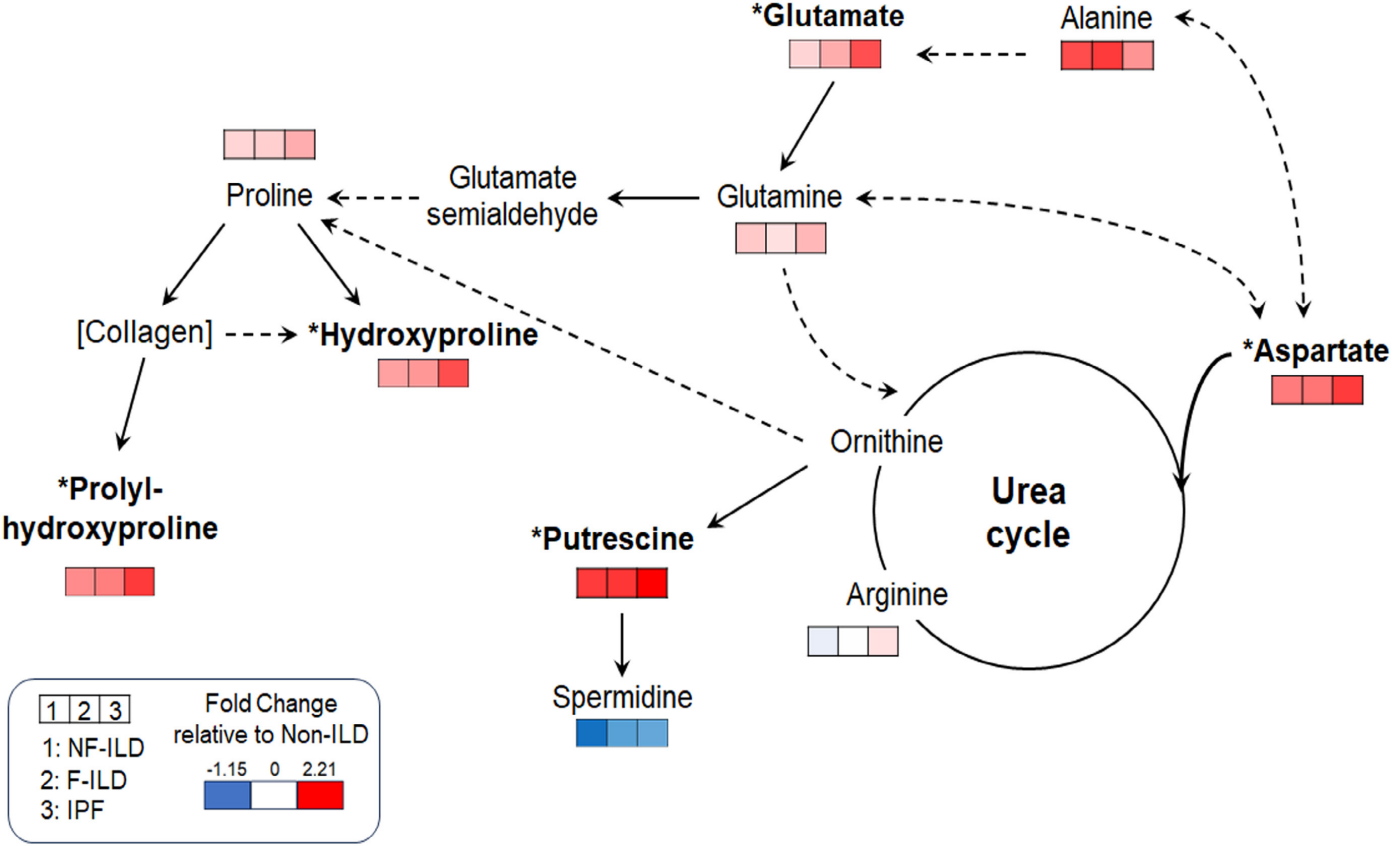
Overview of targeted metabolomics analysis for amino acid‐related metabolites using the human plasma samples. Study groups from left to right: non‐fibrotic ILD (NF‐ILD), fibrotic ILD (F‐ILD), idiopathic pulmonary fibrosis (IPF). Cells were shaded according to the fold change of the mean metabolites' relative intensity in the NF‐ILD, F‐ILD, and IPF groups compared to the non‐ILD group (reference group). The fold changes were calculated by subtracting the mean intensity of the metabolite in the reference group from its mean intensity in each of the other groups. * signifies linear trend *p* ‐value < 0.05

**FIGURE 2:**
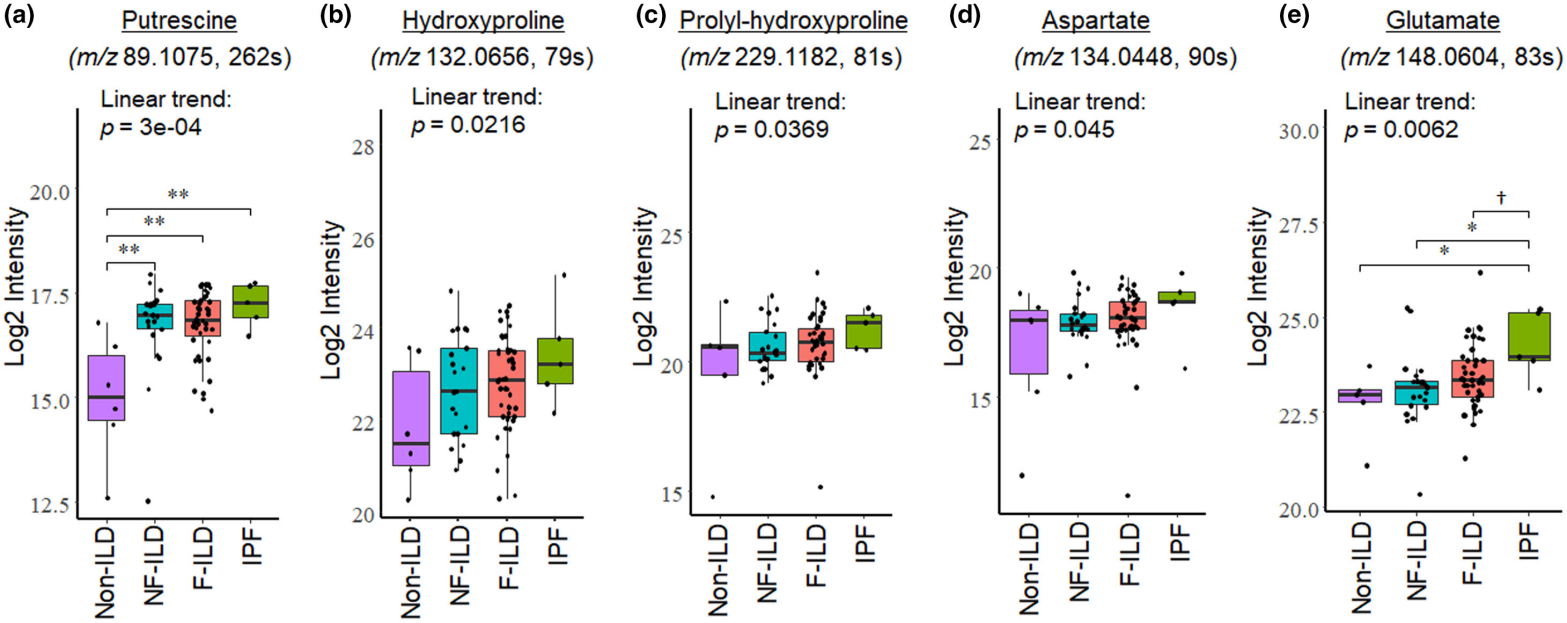
Group comparison analysis of selected metabolites in arginine and proline metabolism. Study groups from left to right: Non‐ILD, NF‐ILD, F‐ILD, IPF, as ordered for linear trend (LT) analyses. *p*‐values of the LT analyses were putrescine (*p* = 0.0003), hydroxyproline (*p* = 0.0216), prolyl‐hydroxyproline (*p* = 0.0369), aspartate (*p* = 0.0450), and glutamate (*p* = 0.0062). To determine pairwise differences between groups, we also tested metabolite abundances by one‐way ANOVA or the Kruskal–Wallis rank sum test followed by either Tukey's honest significant difference test or Dunn's test, respectively, depending on the distribution of each metabolite using the Shapiro–Wilk Test. Significant differences between groups are noted using asterisk notation (†*p* < 0.1; *p < 0.05; **p < 0.01).

Metabolites in arginine and proline metabolism previously described to be altered in pre‐clinical models of pulmonary fibrosis or IPF include: aspartate (Zhao et al., 2017), alanine (Gaugg et al., 2019), arginine (Kitowska et al., 2008; Mora et al., 2006), proline (Durante et al., 2001; Hesse et al., 2001; Kitowska et al., 2008; Pesce et al., 2009), glutamine (Bernard et al., 2018; Cruzat et al., 2018; Ge et al., 2018; Hamanaka et al., 2019), glutamate (Roque & Romero, 2021), creatine (Endo et al., 2003; Zhao et al., 2017), putrescine (Endo et al., 2003; Zhao et al., 2017), spermidine (Endo et al., 2003; Zhao et al., 2017), and hydroxyproline (Endo et al., 2003; Gaugg et al., 2019; Zhao et al., 2017) and prolyl‐hydroxyproline (Endo et al., 2003; Zhao et al., 2017). Given the importance of these metabolites in pulmonary fibrosis, we examined the metabolites involved in arginine and proline metabolism in the present ILD study cohort. We first identified these metabolites in the present study samples using our internal authentic chemical library, MS^2^ database matching, and xMSannotator accordingly (Table 3 and Figure S1), and analyzed the abundance of metabolites among four groups, non‐ILD, NF‐ILD, F‐ILD and IPF (Figures 1 and 2). The results of targeted analysis on putrescine, hydroxyproline, prolyl‐hydroxyproline, aspartate, and glutamate demonstrated significantly increasing linear trends across ILD phenotypes (*p*‐values = 0.0003, 0.0216, 0.0369, 0.0450, and 0.0062 respectively, marked by *bold in Figures 1 and 2), suggesting that the levels of specific metabolites in arginine and proline metabolism change in a linearly trending manner progressing from NF‐ILD to F‐ILD. Our analysis revealed that putrescine levels were higher in the IPF, F‐ILD, and NF‐ILD groups compared to the non‐ILD group (*p*‐values = 0.0028, 0.0048, and 0.0046, respectively, Figure 2). In addition, glutamate levels were higher in the IPF group compared to the F‐ILD, NF‐ILD, and non‐ILD groups (*p*‐values = 0.0750, 0.0120, and 0.0140, respectively, Figure 2). These group comparisons further corroborate the role of these metabolites in ILD fibrometabolism.

**TABLE 3:** Confirmed and annotated ILD‐associated metabolites.

Name	*m/z*	RT, sec	Adduct	HMDB ID	KEGG	Chromatography	MSI confidence level	Schymanski criteria	Identification method^a^
Aspartate	134.0448	90	M + H	HMDB0000191	C00049	HILIC	1	1	*m*/*z*, retention time, MS^2^
Alanine	90.0551	64	M + H	HMDB0000161	C00041	HILIC	1	1	*m*/*z*, retention time MS^2^
Arginine	175.1189	86	M + H	HMDB0000517	C00062	HILIC	1	1	*m*/*z*, retention time, MS^2^
Proline	116.0707	63	M + H	HMDB0000162	C00148	HILIC	1	1	*m*/*z*, retention time, MS^2^
Glutamate	148.0604	83.2	M + H	HMDB0000148	C00025	HILIC	1	1	*m*/*z*, retention time, MS^2^
Glutamine	147.0764	85	M + H	HMDB0000641	C00064	HILIC	1	1	*m*/*z*, retention time, MS^2^
Putrescine	89.1075	262	M + H	HMDB0001414	C00134	HILIC	1	1	*m*/*z*, retention time, MS^2^
Spermidine	146.1652	102	M + H	HMDB0001257	C00315	HILIC	1	1	*m*/*z*, retention time, MS^2^
Hydroxyproline	132.0656	79	M + H	HMDB0000725	C01157	HILIC	1	1	*m*/*z*, retention time, MS^2^
Prolylhydroxyproline	229.1182	81	M + H	HMDB0006695	NA	HILIC	2	2	*m*/*z*, MS^2^ database matching
Creatine	132.0768	64	M + H	HMDB0000064	C00300	HILIC	1	1	*m*/*z*, retention time, MS^2^
Acetamidobutanoate	146.0811	38	M + H	HMDB0003681	C02946	HILIC	1	1	*m*/*z*, retention time, MS^2^
Methylparaben	153.0545	27	M + H	HMDB0032572	NA	HILIC	1	2	*m*/*z*, retention time, MS^2^ database matching
24‐Oxo‐1alpha2325‐trihydroxyvitamin D3	447.3101	29	M + H	HMDB0060129	NA	HILIC	2	4	*m*/*z*

^a^ Schymanski level 1 metabolites were identified in the reference plasma samples using accurate *m*/z signal (±5 ppm from theoretical *m*/*z* for sample *m*/*z*), coelution with authentic standard within 3 s, and MS^2^ spectra matching authentic standard (Liu et al., 2020, Analytical Chemistry). Schymanski level 2 metabolites were identified in the reference plasma samples using accurate m/z signal (±5 ppm from theoretical *m*/z for sample *m*/z) and MS^2^ database matching. Schymanski level 4 metabolites were identified using accurate *m*/z signals (±5 ppm from theoretical *m*/z for sample *m*/z). Prolylhydroxyproline and 24‐Oxo‐1alpha2325‐trihydroxyvitamin D3 were also supported by mummichog and xMSannotator. Methylparaben was supported by xMSannotator.

### 3.3 Untargeted metabolomics analyses

**FIGURE 3:**
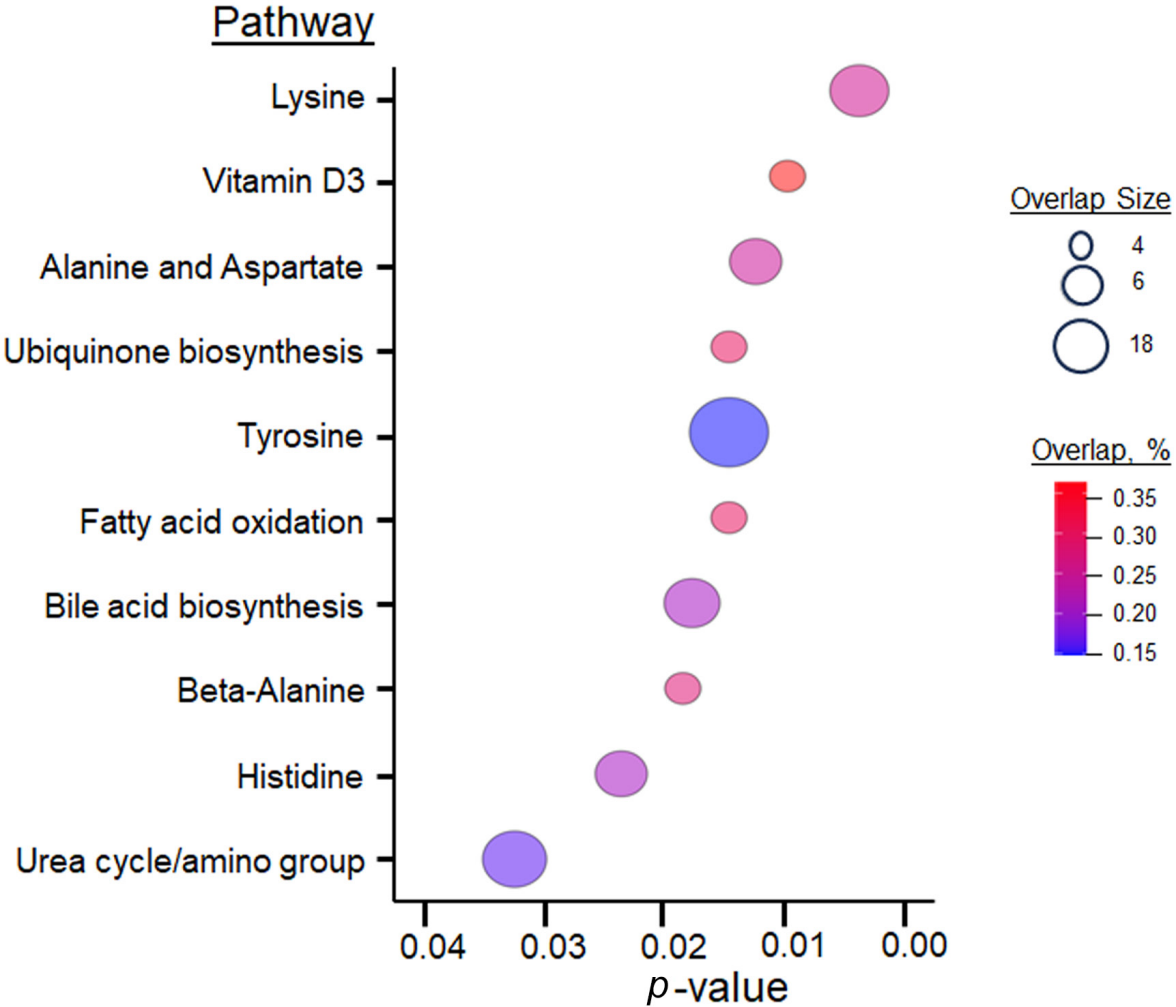
*Mummichog* pathway enrichment analysis of untargeted metabolomics analysis. Bubble plot depicting significantly altered metabolic pathways determined via mummichog pathway enrichment analysis. Bubbles are ordered according to pathway *p*‐value. Pathway overlap size is the number of altered metabolites in each metabolic pathway. The overlap size percentage is the percentage of altered metabolites among the total metabolites in each metabolic pathway.

For untargeted analysis, we used *limma* to identify metabolic features whose abundances differed across four groups, with a *p*‐value of <0.05. Results showed that 715 metabolic features were different in their abundance by ILD phenotypes (Figure 3a). With these 715 metabolic features, we conducted pathway enrichment analysis using the *mummichog* algorithm (Li et al., 2013). The result showed the following altered metabolic pathways are associated with ILD phenotypes: lysine, vitamin D3, alanine and aspartate, ubiquinone biosynthesis, tyrosine, fatty acid oxidation, bile acid biosynthesis, beta‐alanine, histidine, and urea cycle/amino group metabolisms (Figure 3b). These findings support the result of our targeted analyses and provide new insights into additional metabolic pathways that may be involved in ILD fibrometabolism.

**FIGURE 4:**
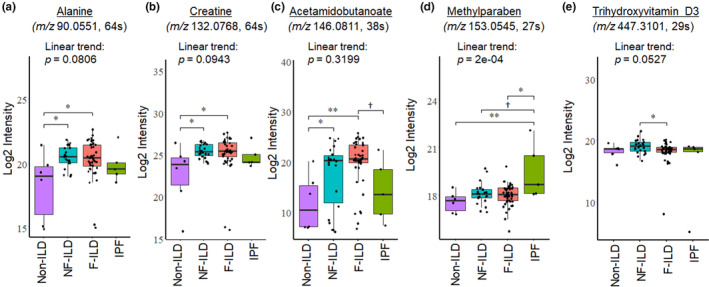
Comparison analysis of selected metabolites from xenobiotics, tyrosine, lysine, urea cycle/amino group, and vitamin D3 metabolism. (a) alanine, (b) creatine, (c) acetamidobutanoate, (d) methylparaben, and (e) trihydroxyvitamin D3 are examined for the linear trend analyses. The following grouping order was used: Non‐ILD, NF‐ILD, F‐ILD, and IPF. *p*‐values of the linear trend (LT) analyses were: Alanine (*p* = 0.0806), creatine (*p* = 0.0943), acetamidobutanoate (*p* = 0.3199), methylparaben (*p* = 0.0002) and trihydroxyvitamin D3 (*p* = 0.0527). To determine pairwise differences between groups, we also tested metabolite abundances by one‐way ANOVA or the Kruskal–Wallis rank sum test followed by either Tukey's honest significant difference test or Dunn's test, respectively, depending on the distribution of each metabolite using the Shapiro–Wilk Test. Significant differences between groups are noted using asterisk notation (†*p* < 0.1; *p < 0.05; **p < 0.01).

For further investigation, we evaluated several critical metabolic pathways determined by high pathway overlap sizes and low *p*‐values, including lysine, vitamin D3 (top 2 low *p*‐values), tyrosine and urea cycle/amino group metabolism (top 2 overlap sizes). We identified alanine, creatine, and acetamidobutanoate in lysine, tyrosine, and urea cycle metabolism (Figure 4 and Table 3). To compare abundance of these metabolites between NF‐ILD, F‐ILD and IPF, we used the same linear trend analysis approach as specified above in the targeted metabolomics analysis. In addition, alanine, creatine and acetamidobutanoate levels were significantly higher in both NF‐ILD and F‐ILD compared to non‐ILD (Figure 4). A xenobiotic metabolite, methylparaben, was found after it was originally annotated as resorcinol monoacetate, an isomer of methylparaben, and found in the tyrosine metabolism. Methylparaben level in IPF was significantly higher than non‐ILD (*p* = 0.0056) and higher than in F‐ILD (*p* = 0.041) (Figure 4, Table 3, and Figure S1). In vitamin D3 metabolism, we identified 24‐Oxo‐1alpha,23,25‐trihydroxyvitamin D3 (trihydroxyvitamin D3), and its level in F‐ILD was significantly lower compared to that in NF‐ILD (*p* = 0.0480) (Figure 4 and Table 3).

## 4 DISCUSSION

Our analysis of plasma metabolites among a variety of clinical phenotypes, non‐ILD, NF‐ILD, F‐ILD, and IPF, revealed significant findings. Our targeted metabolomics approach identified amino acids including aspartate, putrescine, hydroxyproline, prolyl‐hydroxyproline, and glutamate and showed increasing trends across non‐ILD to IPF groups. These amino acids, key in collagen metabolism, suggest a role in pulmonary fibrosis development. Previous studies have shown that a common pathological characteristic of IPF and pulmonary fibrosis is change in amino acid metabolites, including aspartate (Zhao et al., 2017), alanine (Gaugg et al., 2019), arginine (Endo et al., 2003; Kitowska et al., 2008; Mora et al., 2006), proline (Durante et al., 2001; Hesse et al., 2001; Kitowska et al., 2008; Pesce et al., 2009), glutamine (Bernard et al., 2018; Cruzat et al., 2018; Ge et al., 2018; Hamanaka et al., 2019), glutamate (Roque & Romero, 2021), putrescine (Endo et al., 2003; Mora et al., 2006; Zhao et al., 2017), spermidine (Endo et al., 2003; Mora et al., 2006; Zhao et al., 2017), hydroxyproline (Endo et al., 2003; Gaugg et al., 2019; Zhao et al., 2017), prolyl‐hydroxyproline (Endo et al., 2003; Zhao et al., 2017), homocysteine (Seeliger et al., 2022), cystine (Seeliger et al., 2022), and glutamine (Seeliger et al., 2022). Meanwhile, branched‐chain amino acids, including valine, betaine, leucine, and isoleucine were inversely associated with pulmonary fibrosis severity (Seeliger et al., 2022; Summer et al., 2024). Our findings are mostly consistent with the previous studies' findings except that we observed no significant increase of arginine, alanine, glutamine, and spermidine. Collagen metabolism is critical in maintaining lung tissue architecture, where a balance between collagen synthesis and degradation is essential (McKleroy et al., 2013). In pulmonary fibrosis, this balance is disrupted, leading to an accumulation of fibrillar collagen (McKleroy et al., 2013). Recent reports suggest the important role of collagen degradation in regulating the severity of pulmonary fibrosis (McKleroy et al., 2013). Our findings indicate these metabolites could be used as surrogates of increased collagen metabolism and/or impaired degradation and may serve as markers of progression of pulmonary fibrosis not only in IPF, but across a spectrum of fibrosing ILDs.

Building on these insights, our untargeted HRM and pathway enrichment analysis confirmed and expanded upon previous amino acid metabolism findings. We discovered alterations in lysine, vitamin D3, tyrosine, and urea cycle metabolism, all relevant to pulmonary fibrosis. Previous research has demonstrated that the activation of lysine‐specific demethylase 1 contributes to lung myofibroblast differentiation and fibrosis (Pan et al., 2020), and lysyl oxidase promotes bleomycin‐induced pulmonary fibrosis via modulating inflammation (Cheng et al., 2014). Similarly, protein‐derived lysine degradation pathway was altered from mice after the bleomycin administration (Arif et al., 2023). The observed lysine metabolism changes in our study align with these studies, highlighting its role in pulmonary fibrosis. Vitamin D has been linked to pulmonary fibrosis in several studies, including the association between vitamin D deficiency and ILD in human subjects (Hagaman et al., 2011), the induction of pulmonary fibrosis through the renin‐angiotensin system due to chronic vitamin D deficiency in mice (Shi et al., 2017), and the mitigation of pulmonary fibrosis via the deactivation of the lung renin‐angiotensin system after vitamin D supplementation in a mouse model of IPF (Chang et al., 2021). Our data, showing perturbation in vitamin D3 metabolism and trihydroxyvitamin D3 between the NF‐ILD and F‐ILD groups, aligns well with these existing studies. In addition, disruption of tyrosine metabolism was observed with a large enrichment of metabolites in our study. Meanwhile, tyrosine kinase receptors are known to activate signaling pathways linked to pulmonary fibrosis (Allen & Spiteri, 2001; Coward et al., 2010). In line with this, the application of a tyrosine kinase inhibitor, “Nintedanib” mitigated the progression of IPF in phase 3 clinical trials and received the U.S. Food and Drug Administration approval as the first treatment for patients with progressive fibrosing ILD (Richeldi et al., 2014). While our findings indicate altered tyrosine metabolism, it is crucial to distinguish this from the role of tyrosine kinase receptors in the pathogenesis of IPF and pulmonary fibrosis.

A marked increase in methylparaben was observed in linear trend analysis and the IPF groups compared with F‐ILD and non‐ILD groups. Methylparaben is a xenobiotic widely found in cosmetics, personal care products, and medications. Interestingly, methylparaben has been the subject of controversy due to concerns regarding its potential skin irritation and endocrine‐disrupting effects, though the U.S. Food and Drug Administration considers it generally safe when used in low concentration (FDA, 2022). In our study, we found this metabolite increased across all ILD groups, particularly in those with IPF. This suggests that high‐dose exposure to methylparaben from cosmetics, personal care products, and medications, may be a risk factor for IPF.

The presented data are results of a pilot study with a modest sample size in the study groups, meaning limited ability to control for confounders, including age, sex, and smoking history. Additionally, the reference group included was comprised of patients of Emory University Hospital with obstructive pulmonary disease or asthma. While this reference group allowed isolation of metabolic changes associated with progressing fibrosis and ILD, it limited findings related to lung disease in general. Future studies with a larger cohort, including healthy individuals as controls alongside a non‐ILD reference group, will offer better insights for identifying potential biomarkers that distinguish different groups. A hypothesis of this study was that a variety of non‐IPF fibrotic and non‐fibrotic ILD are associated with metabolic changes associated with pulmonary fibrosis as seen in IPF, though to a lesser extent. For this reason, we analyzed these groups in a progressive ordering. Our findings from this pilot study provide new support for this view; however, this is a controversial concept, which does not have a consensus (Wells et al., 2018; Wijsenbeek & Cottin, 2020).

### 4.1 Perspectives and significance

We report that specific metabolites in arginine and proline metabolism including putrescine, hydroxyproline, prolyl‐hydroxyproline, aspartate, and glutamate show increasing linear trends across different ILD phenotypes. These metabolites may reflect progression of pulmonary fibrosis, a condition characterized by a disruption in the balance between collagen synthesis and degradation. Our untargeted metabolomics analysis identified additional altered pathways, including lysine, vitamin D3, tyrosine, and urea cycle metabolism. Methylparaben, a common ingredient in cosmetics, was higher in IPF group, suggesting a potential environmental risk factor. Further research is needed to fully understand the roles of these metabolites in ILD progression and treatment.

### 4.2 Conclusion

In conclusion, despite the small sample size limitation, this study offers a comprehensive metabolic overview of various ILD phenotypes, particularly IPF. It underscores the importance of specific amino acids, metabolic pathways, and xenobiotics in the development of pulmonary fibrosis, guiding future research in this field.

## AUTHOR CONTRIBUTIONS

Study design: X.H., L.M., D.P.J. and Y.M.G. Performance of the experiments: J.Z., J.W., and M.O. Data analysis: J.Z., X.H., and L.M. Manuscript drafting: J.Z. Interpretation of the results, and manuscript review and editing: Z.J., L.M., D.P.J., and Y.M.G.

## FUNDING INFORMATION

This study was supported by National Institute of Environmental Health Sciences grants R01 ES031980 and P30 ES019776, and National Institute of Diabetes and Digestive and Kidney Diseases grant R01 DK125246. This study was also supported by the pilot project funding under P30 ES019776.

## CONFLICT OF INTEREST STATEMENT

The authors declare no competing financial interests.

## DISCLAIMERS

Not eligible.

## ETHIC STATEMENT

The institutional review board of Emory University approved the present study, and we complied with all federal guidelines governing the use of human samples. Plasma samples were collected with informed consent from all subjects under an approved IRB (IRB#00001384).

## Supporting information


**Figure S1:** Ion dissociation spectra (MS^2^) analysis of features identified as methylparaben and prolylhydroxyproline. (a) Ion dissociation spectra (MS^2^) analysis of 153.0545 *m/z*, retention time 27 s (hcd 35) in the plasma sample from a IPF patient. (b) Ion dissociation spectra (MS^2^) analysis of 229.1182 *m/z*, retention time 81 s (hcd 35) in the plasma sample from a IPF patient.

## Data Availability

The data are available within the article, supplementary information, or available from the authors upon reasonable request. De‐identified raw data files are available on the Metabolomics Workbench.
