# Supplementary figures and images for: A pilot metabolomics study across the continuum of interstitial lung disease fibrosis severity
Source: Physiol Rep. 2024 Oct 18;12(20):e70093. doi: 10.14814/phy2.70093 (PMC11489002; doi:10.14814/phy2.70093)

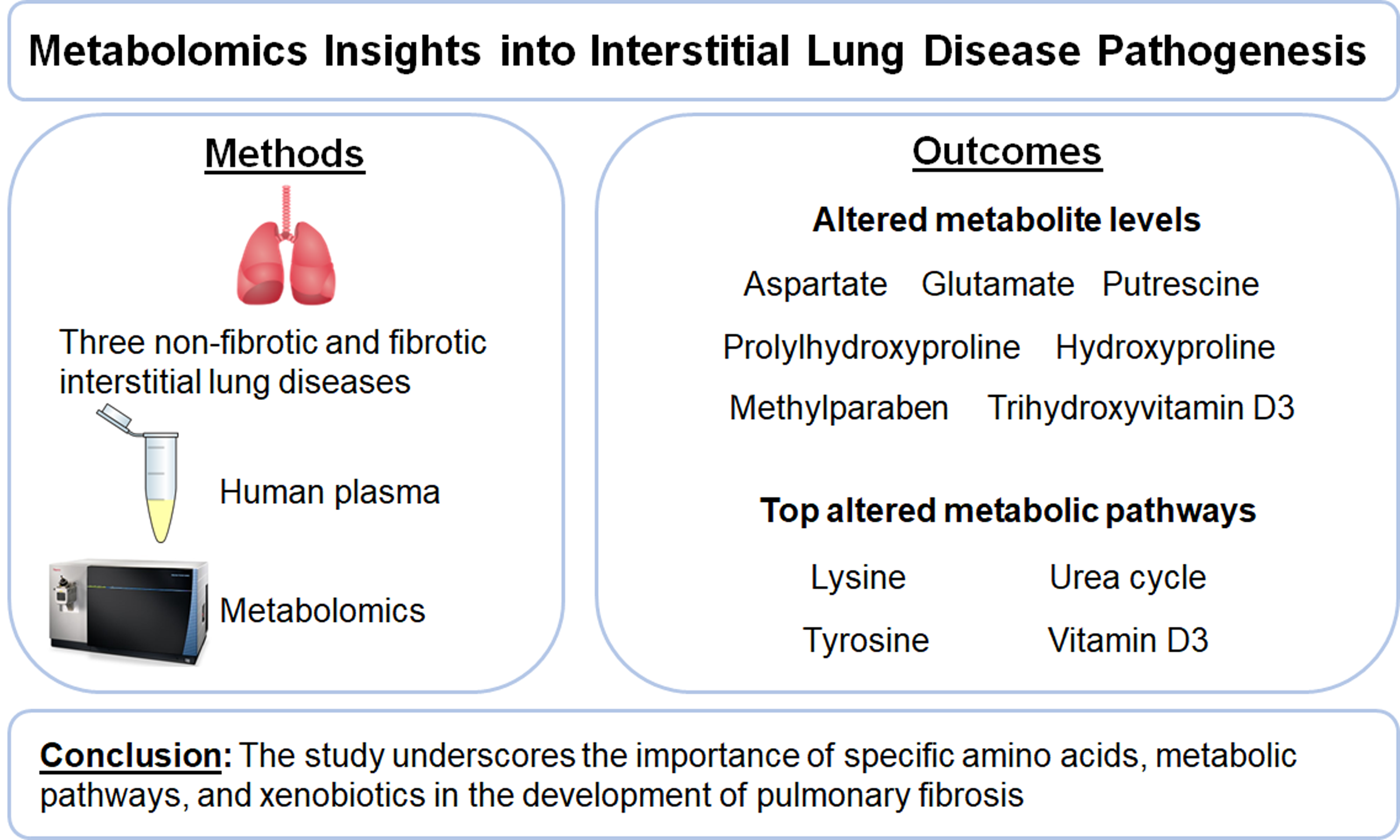

Supplement: Supplementary file 1 — Figure S1: Ion dissociation spectra (MS2) analysis of features identified as methylparaben and prolylhydroxyproline. (a) Ion dissociation spectra (MS2) analysis of 153.0545 m/z, retention time 27 s (hcd 35) in the plasma sample from a IPF patient. (b) Ion dissociation spectra (MS2) analysis of 229.1182 m/z, retention time 81 s (hcd 35) in the plasma sample from a IPF patient. [file PHY2-12-e70093-s001.tif]
